# Non-Toxic Gold Nanoclusters for Solution-Processed White Light-Emitting Diodes

**DOI:** 10.1038/s41598-018-27201-x

**Published:** 2018-06-11

**Authors:** Yu-Chiang Chao, Kai-Ping Cheng, Ching-Yi Lin, Yu-Li Chang, Yi-Yun Ko, Tzu-Yin Hou, Cheng-Yi Huang, Walter H. Chang, Cheng-An J. Lin

**Affiliations:** 1 Department of Physics, National Taiwan Normal University, Taipei, 11677 Taiwan; 2 Department of Physics and Center for Nanotechnology, Chung Yuan Christian University, Chung-Li, 32023 Taiwan; 3 Department of Biomedical Engineering and Center for Biomedical Technology, Chung Yuan Christian University, Chung-Li, 32023 Taiwan

**Keywords:** Electronic devices, Organic LEDs

## Abstract

Solution-processed optoelectronic devices are attractive because of the potential low-cost fabrication and the compatibility with flexible substrate. However, the utilization of toxic elements such as lead and cadmium in current optoelectronic devices on the basis of colloidal quantum dots raises environmental concerns. Here we demonstrate that white-light-emitting diodes can be achieved by utilizing non-toxic and environment-friendly gold nanoclusters. Yellow-light-emitting gold nanoclusters were synthesized and capped with trioctylphosphine. These gold nanoclusters were then blended with the blue-light-emitting organic host materials to form the emissive layer. A current efficiency of 0.13 cd/A was achieved. The Commission Internationale de l’Eclairage chromaticity coordinates of (0.27, 0.33) were obtained from our experimental analysis, which is quite close to the ideal pure white emission coordinates (0.33, 0.33). Potential applications include innovative lighting devices and monitor backlight.

## Introduction

Chemically synthesized colloidal semiconductor quantum dots exhibit size-dependent properties as their diameter is smaller than the Bohr exciton radius^1^. The energy gap, absorption spectra, and optical emission characteristics of the quantum dots are all influenced by their size because of the strong quantum-confinement effect. The property of tunable optical absorption and emission makes colloidal semiconductor quantum dots broadly applicable in fields such as biology^2,3^, solar cells^4,5^, and light-emitting diodes (LEDs)^6–14^. Device structures of early quantum dot LEDs were similar to those of polymer LEDs, comprising a blend of quantum dots and a polymer as the emissive layer^10,11^. To achieve monochromic emission, the electroluminescence from the polymer had to be diminished; therefore, quantum dot LEDs in layered architectures were developed with a monolayer of quantum dots sandwiched between the hole and electron transport layers^6–9^. Narrow emission spectra of quantum dot LEDs were successfully realized throughout the visible^6–9^ and near-infrared spectrum^12^, and it has been demonstrated that quantum dot LEDs are excellent candidates for next-generation display^6,7^. In addition, for solid-state lighting applications, white-light-emitting quantum dot LEDs have been realized^13,14^. A mixed-monolayer of red, green, and blue light-emitting quantum dots were used for the active layer, and the ratio of the different color quantum dots was precisely controlled to generate white light^13^. Besides, white light emission can be achieved in a blend film of quantum dots and polymeric materials by tuning the concentration ratio of the quantum dots/polymer blend^14^. Through controlling the Föster energy transfer between the quantum dots and polymer, white light output was achieved by mixing the emission from the quantum dots and polymeric materials. However, the utilization of toxic elements such as lead^2,6,7,12^ and cadmium^2,6,8–11,13,14^ in current optoelectronic devices on the basis of colloidal quantum dots raises environmental concerns over its potential for massive industrial production and subsequent hazardous disposal^15^. Research on non-toxic alternatives with comparable optoelectronic properties is therefore essential^16–18^.

In contrast to semiconducting quantum dots as fluorescent probes, fluorescent noble metal nanoclusters (NCs) are becoming newly biological labels with no toxic heavy metals^19,20^. Noble metal (e.g., Au, Ag) NCs, with sizes comparable to the Fermi wavelength of an electron, show molecule-like behaviors such as discrete electronic states and strong fluorescence. The emission spectra of these noble metal NCs can be modulated through the selection of capping molecules and the control of their size (diameter)^19^. A simple spherical jellium model, *E*_Fermi_/*N*^1/3^, has been developed to correlate the number of atoms per cluster (N) and the Fermi energy of bulk gold (*E*_Fermi_) with the emission spectrum^21^. Because of the non-toxic nature and the broad size-dependent emission range from visible to near-infrared^19^, noble metal NCs demonstrate great potential for use in optoelectronic devices. Electroluminescence from metal (Au^22^, Ag^23^, and Cu^24^) NCs with mechanically or electrically induced nanoscale junctions has been demonstrated; however, reproducibility and application of such molecular electronics for realizing nanoscale junctions remains a concern^25^. LEDs based on metal nanoclusters have been demonstrated with emission from orange to infrared^26,27^.

In this study, solution-processed white-light-emitting Au NC-LEDs were fabricated and tested for the first time based on an emissive layer of yellow-light-emitting Au NCs (serving as a dopant) with blue-light-emitting poly(N-vinyl carbazole) (PVK) as the host. The hole-transporting N,N′-Bis(3-methylphenyl)-N,N′-diphenylbenzidine (TPD) and electron-transporting 2-(4-tert-Butylphenyl)-5-(4-biphenylyl)-1,3,4-oxadiazole (PBD) were also blended with the PVK to improve charge transport in the emissive layer. White electroluminescence was achieved by mixing the yellow-light-emitting Au NCs and the blue-light-emitting host. A current efficiency of 0.13 cd/A was obtained. The Commission Internationale de l’Eclairage (CIE) chromaticity coordinates of the Au NC-LED color are (0.27, 0.33), which are very close to that of the ideal pure white emission coordinates (0.33, 0.33). The white-light-emitting Au NC-LEDs demonstrated in this study were fabricated by solution processes that have been widely used for conventional quantum dot and polymer LEDs, making the fabrication of Au NC-LEDs easy and providing the possibility for large-scale production.

White light can be generated from a single device unit through mixing the electroluminescence from two complementary colors (blue and orange) or three primary colors (red, green, and blue). One approach to achieve white-light-emitting devices is to use a multi-layer device structure stack with several layers emitting different colors^28,29^. Another approach involves the blending of emissive materials in a single emission layer. White light emission has been successfully realized with a single emission layer, either by blending a small amount of red-light-emitting polymer^30,31^ or small-molecular weight emitter molecules^32^ into a blue-light-emitting host polymer. Since the fabrication of devices with a single emissive layer is the most straightforward way to generate white light, we adopt this simplest device structure in this study to demonstrate the idea of white-light-emitting Au NC-LEDs. In this study, the synthesized yellow-light-emitting Au NCs were blended with the blue-light-emitting PVK, TPD, and PBD host materials to prepare the emissive layer.

## Materials and Methods

### Device Fabrication

The light-emitting devices were fabricated on indium tin oxide (ITO) glass substrates. The ITO glass substrates were cleaned sequentially by acetone, isopropanol, and DI water, respectively. After UV-ozone treatment for 30 min, the hole transport material poly(3,4-ethylenedioxythiophene):poly(styrene sulfonate) (PEDOT:PSS, Clevios P VP AI4083) was spin-coated onto the substrates and baked at 120 °C for 20 min. The substrates were then transferred to a N_2_-filled glovebox for the following fabrication processes. A blend solution consisting of poly(N-vinyl carbazole) (PVK), 2-(4-tert-Butylphenyl)-5-(4-biphenylyl)-1,3,4-oxadiazole (PBD), N,N′-Bis(3-methylphenyl)-N,N′-diphenylbenzidine (TPD), and Au NCs was spin-coated on the substrates to form an emissive layer about 70 nm. The Au NCs were synthesized with the assist of ultrasonic irradiation. Gold trichloride powders (37.5 mg AuCl_3_, ACROS) were prepared within the 8-mL glass vial and then add 5 mL of Toluene to form a turbid solution. After precipitating all insoluble parts by centrifugation, the supernatant appear in blue-emitted fluorescence. The yellow emitted gold nanoclusters can be obtained by introducing the ultrasound energy. The as-prepared turbid AuCl_3_/Toluene mixture was treated by ultrasound sonication (MISONIX with microtip, 20 kHz, 120 W, and Total process time: 30 mins (5 seconds ON, 1 second OFF)). After cooling to room temperature and removing the agglomerates (3000 r.p.m, 5 mins), 50 μL of trioctylphosphine (TOP, 0.5 M in toluene) was added to stabilize the nanoclusters and resulted in bright yellow-emitted gold nanoclusters under UV irradiation. The solution for preparing emissive layer was prepared by blending a mixture of PVK, PBD, and TPD in chlorobenzene (100 ul) with a solution of TOP-capped Au NCs (300 ul). The weight blending ratio is PVK:PBD:TPD = 61:24:9. After annealing substrates at 120 °C for 20 min, 1.2 nm LiF and 70 nm Al were finally deposited as cathode to complete the devices.

### Measurement and Characterization

Electrical characteristics were measured by a Keithley 2400 instrument. The luminance of the devices was measured by a luminance meter (LS-110, Konica Minolta). Film thicknesses were characterized by field-emission scanning electron microscope (FESEM, JEOL JSM-7600F). The absorbance and fluorescence of Au NCs were analyzed by UV-vis absorption spectroscopy (Cary 50, Varian, USA) and PL spectroscopy (Fluoromax-3, Horiba). The absorbance spectra of the solid films and the electroluminescence spectra of the devices were recorded with a microspectrometer (SD1200‐LS‐HA, StreamOptics Co.). The thickness of organic layers are monitored by ET200 (Kosaka Laboratory Ltd.).

## Results and Discussion

**Figure 1:**
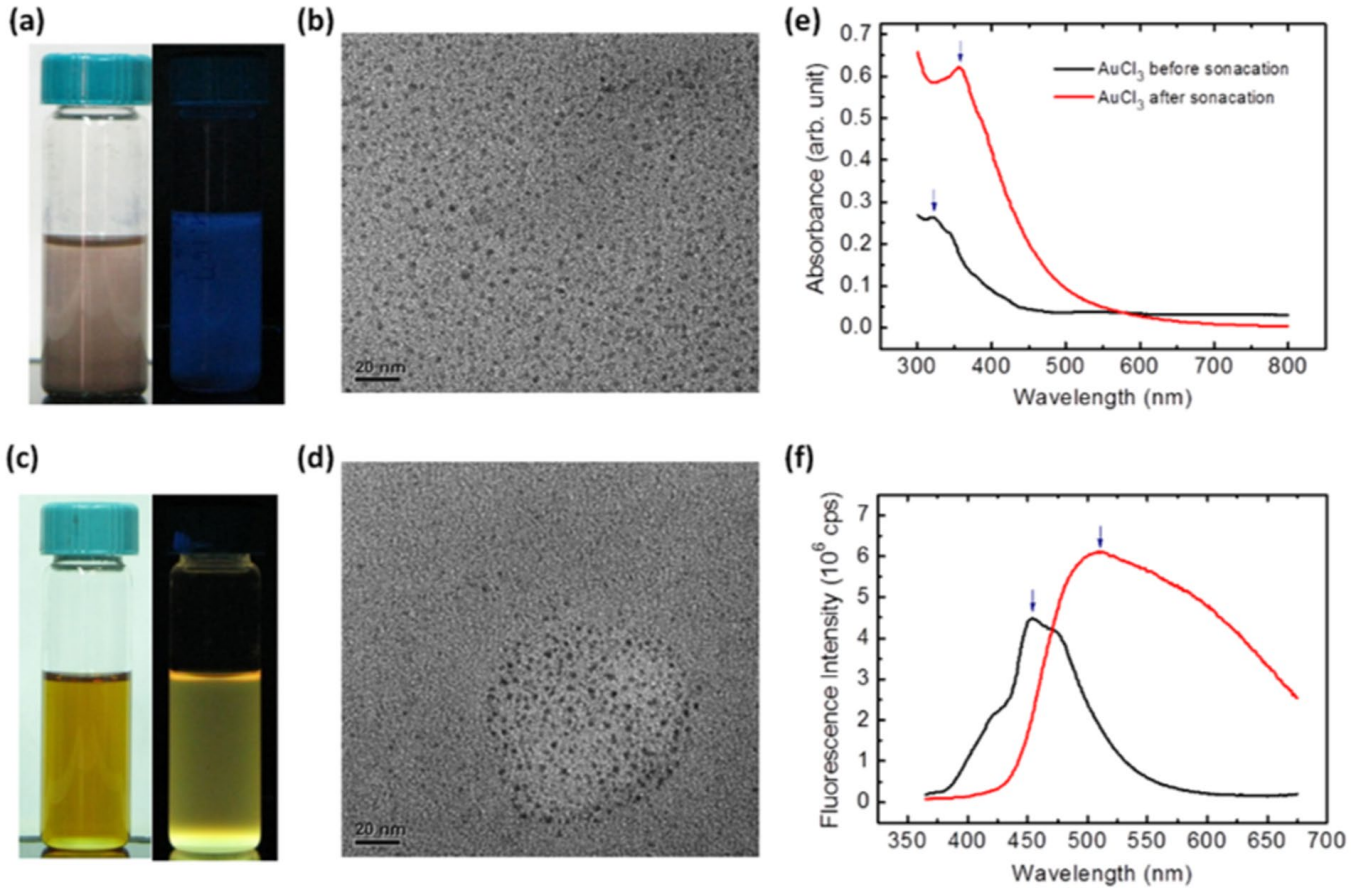
Images and photophysical response of the Au NCs. The as-prepared AuCl3/toluene mixture contains soluble blue-emitting Au NCs which were shown under (**a**) daylight and UV illumination and (**b**) TEM. Yellow-light-emitting Au NCs can be further obtained by ultrasound sonication as well as TOP stabilization, which are again shown under (**c**) daylight and UV illumination and (**d**) TEM. The corresponding UV-VIS absorbance and the photoluminescence spectra of the Au NCs are also shown in (**e**) and (**f**).

The synthesis of Au NCs was modified from our previous sonochemical method^33^. Briefly, blue-light-emitting Au NCs can be rapidly formed by dispersing the anhydrous powder of gold (III) chloride (AuCl_3_) in toluene without sonication (Fig. 1a). The absorbance spectrum of the Au NCs showed an absorption peak at ~320 nm and intense blue fluorescence at 450 nm (Fig. 1e,f). From the TEM micrograph in Fig. 1b, the blue-light-emitting Au NCs are too small to possess the continuous density of states and surface plasmon resonance^19^. These optical characteristics are consistent with those mentioned in a previous report, which demonstrates that the most abundant composition is Au_16_^33^. The photoluminescence (PL) spectrum of the Au NC ranges from 400 to 550 nm and shows a peak at ~450 nm. However, the blue-light-emitting Au NCs are not suitable for blending with blue-light-emitting PVK to produce white light emission. Yellow-light-emitting Au NCs can be further synthesized by treating the NC solution with high-powered ultrasound. Upon capping with trioctylphosphine (TOP), bright yellow-emitting Au NCs not only showed a featured absorption peak at about 360 nm (Fig. 1e) but also covered a broad PL spectrum from 450 to 700 nm (Fig. 1f). The yellow-emission of the TOP-capped Au NCs was observed under daylight and UV light illumination (Fig. 1c) and the TEM micrograph of the yellow-light-emitting Au NCs show the size to be smaller than 2 nm on average (Fig. 1d). The mass spectra, photoluminescence excitation, photoluminescence lifetime, and photostability are shown in Figs S1 and S2. After TOP surface modification, the photostability is enhanced. Fluorescence intensity decreases 30% and 13% for Au NCs without and with TOP surface modification, respectively. PL quantum yield is also enhanced after TOP surface modification. PL quantum yields for Au NCs before and after TOP surface modification are 3.4% and 4.99%. The PL decay curve of Au NCs with TOP surface modification shows a fast and a slow component, and can be fitted with a bi-exponential decay function. The short-lived PL lifetime is 5.6 ns, while the long-lived PL lifetime is 32.9 ns. Yellow emission is now achieved, which is suitable for blending with the blue-light-emitting PVK to generate white light. The change in these optical characteristics is attributed to the sizable highest occupied molecular orbital (HOMO)-lowest unoccupied molecular orbital (LUMO) energy gap generated from the molecular modification^34,35^.

**Figure 2:**
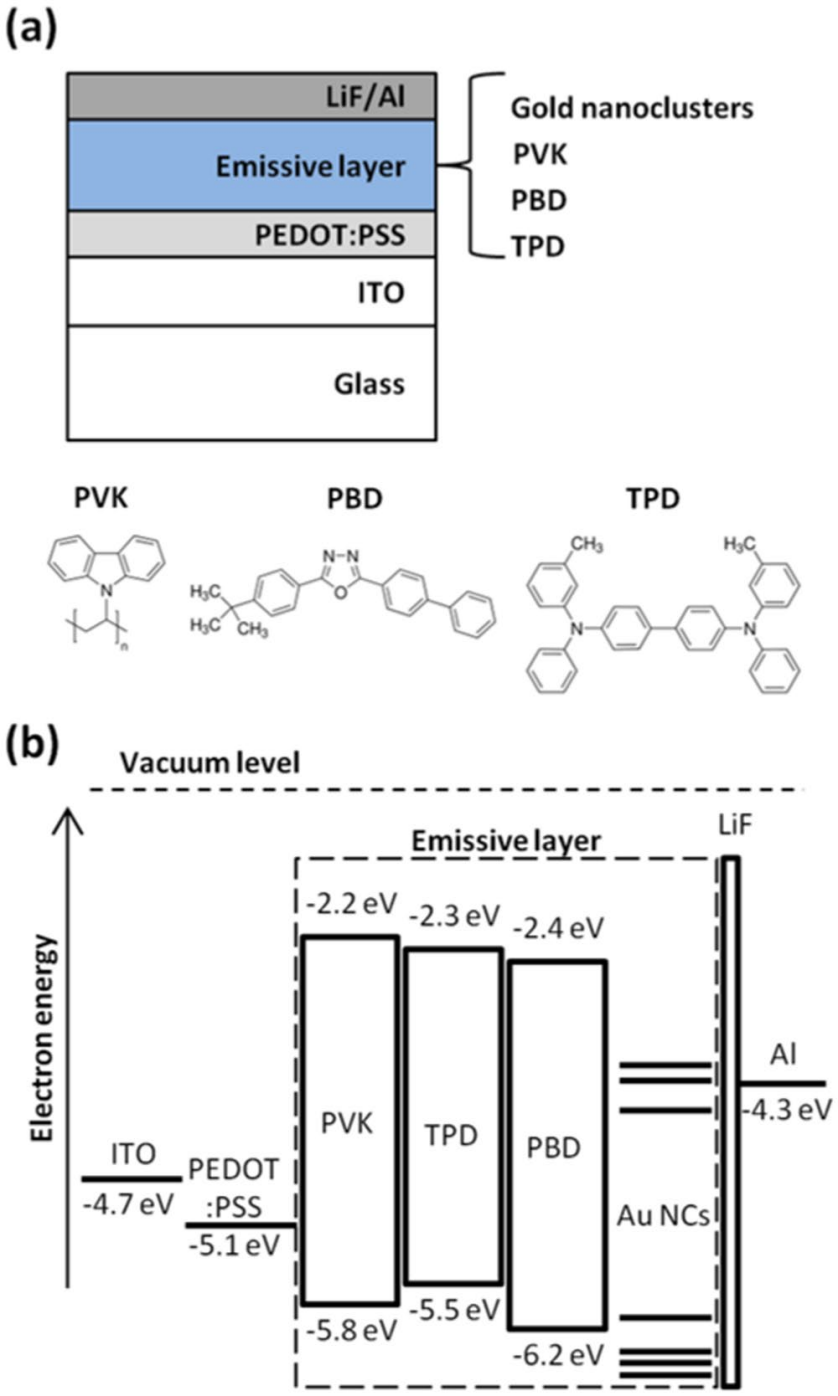
Device structure and band diagram. (**a**) Schematic device structure and (**b**) band diagram of the white-light-emitting diode based on gold nanoclusters. The molecular structures of the host materials are also shown in (**a**).

**Figure 3:**
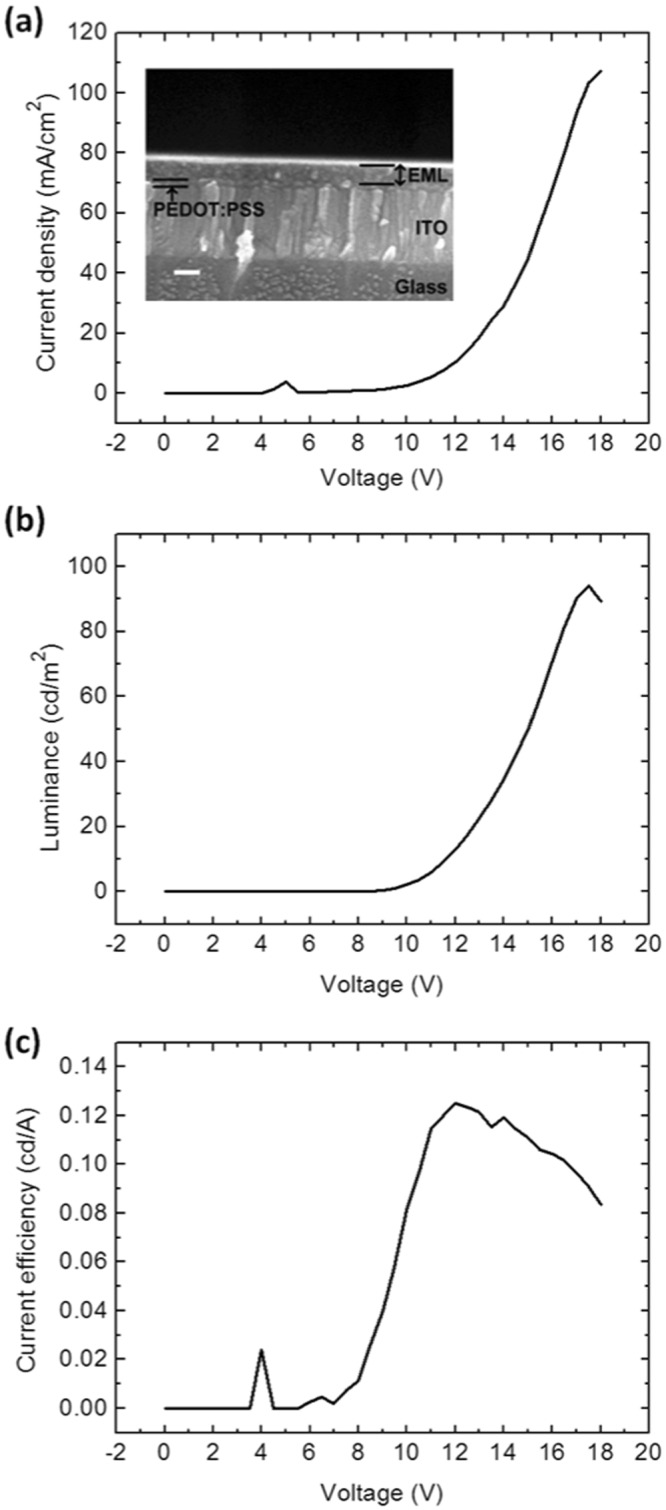
Device performance of the white-light-emitting diodes based on gold nanoclusters. (**a**) The current density, (**b**) luminance, and (**c**) current efficiency are plotted as a function of voltage. The inset in (**a**) is the cross-sectional SEM image of the emissive layer. The scale bar represents 100 nm.

The schematic device structure of the heavy metal-free and white-light-emitting Au NC-LEDs is shown in Fig. 2a. The poly(3,4-ethylenedioxythiophene):poly(styrene sulfonate) (PEDOT:PSS) combination was used as the buffer layer to flatten the surface of indium tin oxide (ITO) and to increase the anode work function. Lithium fluoride (LiF) and aluminum (Al) were used for the cathode. The 70 nm emissive layer was prepared by spin coating the blend solution of PVK, PBD, TPD, and TOP-capped Au NCs, as shown in the inset of Fig. 3a. The emissive layer is uniform and reproducible since similar thickness can be obtained from different substrates and no remarkable objects can be observed far from the edges for SEM inspection (Fig. S3). The molecular structures of PVK, PBD, and TPD are shown in Fig. 2a. The HOMO level, the LUMO level and the work functions of these materials are shown in the band diagram of the white-light emitting Au NC-LEDs (Fig. 2b)^36^. The molecular orbital energy level diagram of the Au NC is adopted from the literature and shown in Fig. 2b^37–40^. Time-dependent density functional theory calculations were performed for the electronic structure^37^. The highest occupied molecular orbital and the lowest unoccupied molecular orbital are predicted to be mainly composed of 6sp atomic orbitals, and the predicted absorption spectrum and electronic structure are quite well matched^37^. A widely used weight blending ratio was selected to be PVK:PBD:TPD = 61:24:9 (ref.^36^) because the hole mobility of TPD (μ_h_ ~ 1 × 10^−3^ cm^2^ V^−1^ s^−1^) (ref.^41^) and the electron mobility of PBD (μ_e_ ~ 2 × 10^−5^ cm^2^ V^−1^ s^−1^) (ref.^42^) are higher than that of PVK (μ_h_ ~ 1 × 10^−9^ cm^2^ V^−1^ s^−1^ and μ_e_ ≪ μ_h_) (ref.^36^). The PVK was blended with TPD and PBD to facilitate the hole and electron transport, respectively. Besides, because of the higher HOMO level of TPD and lower LUMO level of PBD in comparison to the corresponding levels of PVK, the addition of TPD and PBD also resulted in better hole and electron injection.

Figure 3 shows the device performance of the white-light-emitting Au NC-LED. A rectifier characteristic was observed from the current density–voltage curve, as shown in Fig. 3a. This is because the injection barriers for both electrons and holes are high when the device is reverse biased, while the electron and hole injection barriers are low when the device is forward biased. The luminance–voltage curve shown in Fig. 3b demonstrates that a luminance of 1 cd/m^2^ was obtained when the device was biased at 9.5 V. The luminance increased with increasing bias and showed a maximum value of ~100 cd/m^2^ at a bias voltage of 18 V. The current efficiency of the white-light-emitting Au NC-LEDs was higher than 0.1 cd/A for a voltage bias range of 10 ~ 17 V, as shown in Fig. 3c. A maximum current efficiency of ~0.13 cd/A was obtained at a bias voltage of 12 V.

**Figure 4:**
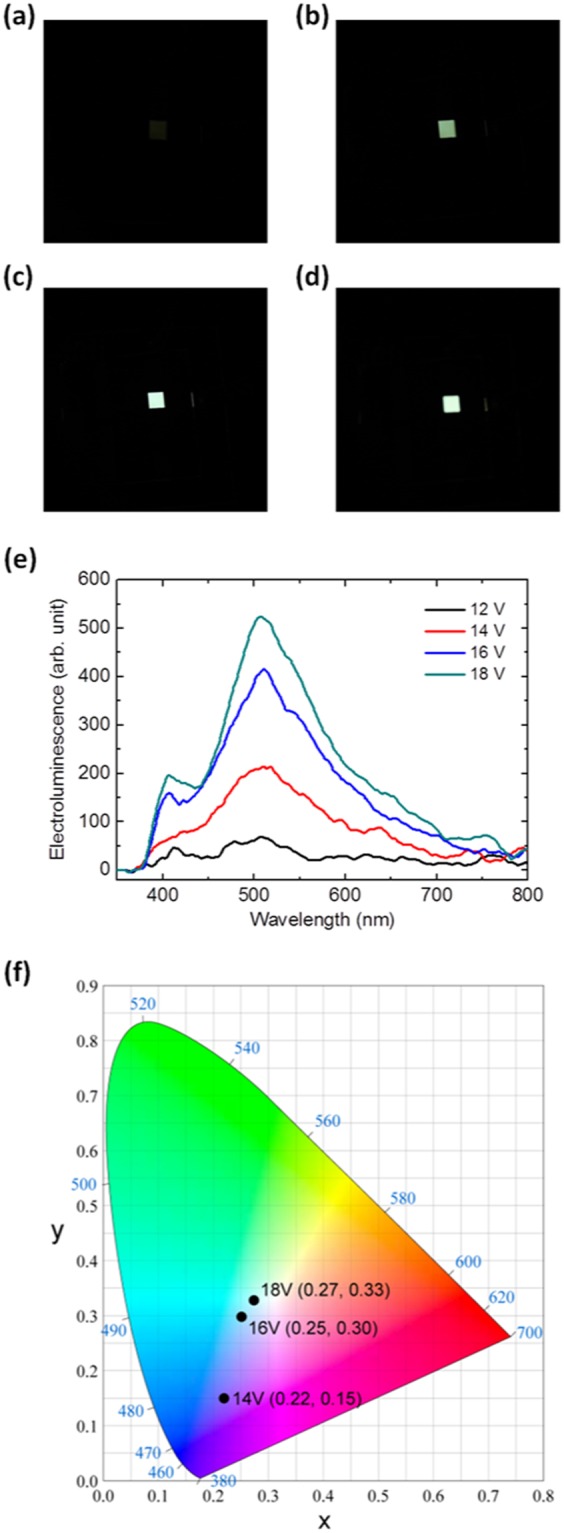
Device optical performance. (**a**–**d**) Photographs of a device under 12, 14, 16 and 18 V. (**e**) Electroluminescence spectra and (**f**) the CIE coordinates of the white-light-emitting Au NC-LED under various bias conditions.

The photographs, electroluminescence (EL) spectra, and the CIE coordinates of a white-light-emitting Au NC-LED, under various bias conditions, are shown in Fig. 4. The Au NC-LED showed higher EL intensity when larger bias voltage was applied (Fig. 4a–e). The EL spectrum of the Au NC-LED biased at 14 V resembled the PL spectrum of the solution of TOP-capped Au NCs (Fig. 1f), whereas the contributions of the blue-light-emitting host materials were relatively low. However, with rising bias voltage, two spectral maxima were revealed in the EL spectrum. A pronounced EL peak at ~410 nm was observed in both of the EL spectra for the Au NC-LED, biased at 16 and 18 V. The CIE coordinates for an Au NC-LED under various biases are indicated in the chromaticity diagram, as shown in Fig. 4f. When the device was biased at 14 and 16 V, CIE chromaticity coordinates of (0.22, 0.15) and (0.25, 0.30) were obtained, respectively. When the bias voltage was increased up to 18 V, the CIE chromaticity coordinates of (0.27, 0.33) were obtained, which were very close to the ideal coordinates of pure white emission (0.33, 0.33). The white LEDs have been achieved based on Au NCs. Comparing with the device without Au NCs which shows EL peak at 480 nm, as shown in Fig. S4a, the peak of the EL spectrum of the device with Au NCs is red shifted to ~510 nm, which is also the PL peak of the Au NCs (Fig. 1f). Besides, when various voltage biases were applied, the peaks of all the EL spectra of the device without Au NCs locate at 480 nm (Fig. S4b), while those peaks locate at 510 nm for device with Au NCs (Fig. 4e). The color of the device without AuNCs is blue under various biases. All these results further indicate that the Au NCs indeed contribute to the EL spectrum.

Eleven devices were fabricated in order to understand the reproducibility of the Au NCs LEDs. As shown in Fig. S5, all devices function well with performance variations. The photographs of the other five devices under forward biases were also shown in Fig. S6. The best current density and external quantum efficiency of a device falls in the range between 0.04~0.18 cd/A and 0.02~0.08%, respectively. The color rendering index is about 78, while the color temperature is about 7100 ~ 7500 K. Since the current efficiency varies in one order of magnitude, we believe that the Au NCs LEDs is reproducible.

Above mentioned characteristics are obtained from fresh devices. Now we turn to understand the stability of the device during multiple measurements. As shown in Fig. S7, one sample was measured for four times sequentially. The Au NCs LED shows a decrease in current density and luminance when we measure the same device again. However, the changes in current density and luminance were not much. Besides, current efficiency was similar. The bump observed at about 4 V will disappear after measuring the same device for many times. This pump is attributed to some short circuit paths between electrodes and will be burned down after operation. All these observations are similar to the organic LEDs which usually show a slight decrease in device performance when monitoring the characteristics of a fresh device with time. However, long term stability for Au NCs LEDs is still an important issue which should be investigate in the future.

The operation of the white-light-emitting Au NC-LEDs is described below. When the device is subjected to a forward bias, with ITO positively biased with respect to Al, holes are injected from the ITO contact through the PEDOT:PSS layer into the emissive layer, and are transported to the Au NCs. Similarly, electrons are injected from the LiF/Al cathode into the emissive layer and are transported to the Au NCs. The exciton generation can be achieved via charge trapping, Föster energy transfer from the host to the Au NCs, or through interfacial exciplex states. In the charge trapping case, electrons might be trapped in the Au NCs, owing to the relative energy alignment of the LUMO levels of the host materials and Au NCs. Thus, efficient exciton formation on the Au NCs can occur after recombination with the holes. In the Föster energy transfer case, excitons form on host materials and undergo Föster energy transfer to the Au NCs, where excitons might decay radically. The PVK, PBD, and TPD are materials with pronounced absorption below 400 nm and PL at ~410 nm^43–46^. Since the absorbance spectrum of TOP-capped Au NCs (Fig. 1e) overlaps with the PL spectra of PVK, PBD, and TPD to a reasonable extent, Föster energy transfer is enabled from the host to the TOP-capped Au NCs. In the interfacial exciplex state case, holes in the HOMO of organic materials and the electrons in the Au NCs might combine to form exciplex states at the interface between the organic and Au NC^47^. The possibility of the exciplex at the organic-Au NC interface cannot be ruled out because the PL spectrum of the solution of TOP-capped Au NCs and the EL spectrum of the Au NC-LED biased at high voltage do not perfectly match. The exciplex at the organic-Au NC interface might undergo radiative recombination to produce yellow light according to the energy band diagram shown in Fig. 2b. However, the relative contributions of charge trapping, Föster energy transfer, and interfacial exciplex states still remain to be explored in future studies. No matter which mechanism is the dominant process in the white-light-emitting Au NC-LEDs, the dual observation of emission from the blue host and yellow TOP-capped Au NCs in the EL spectrum (Fig. 4e) is indicative of the incomplete charge trapping or energy transfer phenomena. The mixing of the blue and yellow light from the Au NC-LEDs successfully yields white-light emission. The voltage-dependent color and EL spectrum might result from the field-dependent competition between charge trapping and unperturbed charge transport^48^. Since the trapping rate of carrier depends on the electric field, the proportion of the emission from Au NCs is related to the applied field and the contribution from the Au NCs will be increased with increasing the voltage bias. Our findings shown in Fig. 4e indeed indicate the increase contribution from Au NCs which makes the possibility of above-mentioned mechanism high. Besides, the increasing contribution of the broad PL spectrum of the Au NCs with increasing voltage bias leads to a voltage-dependent CIE coordinates, as shown in Fig. 4e.

Early reports of white quantum dot LEDs shown external quantum efficiency of 0.36% and color rendering index of 86^13^. Recently, superior white device performance was reported with peak luminescence of 23,000 cd/m^2^, current efficiency of 21.8 cd/A and external quantum efficiency of 10.9%^49^. Although the current efficiency of the newly-developed white-light-emitting Au NC-LEDs is lower than that of the state-of-the-art quantum dot and polymer LEDs, the current efficiency of Au NC-LEDs is still higher than the efficiency of other competent LED technologies at their early development stage^10,11,50^. This is indeed a good start for the newly-developed white-light-emitting Au NC-LED. The main cause for the low performance of Au NC-LEDs may come from the non-optimized surface modification on Au NCs. It has been reported in a recent review paper^32^ that surface modification has a significant influence on the emission wavelength and the quantum yield. It is expected that the device performance can be further enhanced by choosing a better capping molecule on the surface of Au NCs. Besides, in current device structures, no hole transport layer/electron blocking layer or electron transport layer/hole blocking layer was used that can confine the exciton to within the emissive layer. To further enhance the performance of white-light emitting Au NC-LEDs, a hole transport layer/electron blocking layer might be added between the PEDOT:PSS and emissive layers, and an electron transporting layer/hole blocking layer might be inserted between the emissive layer and the cathode. In such device geometry, due to the formation of an energy barrier for the holes or electrons, excitons can be confined well to stay within the emissive layer without being quenched by the electrodes, thereby causing the device efficiency to be enhanced. Furthermore, adding an electron injection layer, such as 8-hydroxyquinolinolato lithium, between electron transport layer and cathode is helpful to reduce the electron injection barrier.

## Conclusion

The yellow-light-emitting TOP-capped Au NCs were synthesized and blended with the blue-light-emitting host material to realize white-LEDs in a device structure that resembles quantum dots and polymer LEDs. We successfully demonstrated the white-LEDs based on non-toxic Au NCs with a current efficiency of 0.13 cd/A and CIE chromaticity coordinates of (0.27, 0.33). The external quantum efficiency is estimated to be 0.04 at 18 V. This realization of white-light-emitting Au NC-LEDs, for the first time, might lead to new environment-friendly Au NC-based optoelectronic devices in the near future for photonic technology application.

## Electronic supplementary material

Supplementary Information
