# Publisher Correction: Non-Toxic Gold Nanoclusters for Solution-Processed White Light-Emitting Diodes

**DOI:** 10.1038/s41598-019-43788-1

**Published:** 2019-05-17

**Authors:** Yu-Chiang Chao, Kai-Ping Cheng, Ching-Yi Lin, Yu-Li Chang, Yi-Yun Ko, Tzu-Yin Hou, Cheng-Yi Huang, Walter H. Chang, Cheng-An J. Lin

**Affiliations:** 1 Department of Physics, National Taiwan Normal University, Taipei, 11677 Taiwan; 2 Department of Physics and Center for Nanotechnology, Chung Yuan Christian University, Chung-Li, 32023 Taiwan; 3 Department of Biomedical Engineering and Center for Biomedical Technology, Chung Yuan Christian University, Chung-Li, 32023 Taiwan

Correction to: *Scientific Reports* 10.1038/s41598-018-27201-x, published online 11 June 2018

In the original version of this Article, Yu-Li Chang, and not Cheng-An J. Lin, was incorrectly listed as a corresponding author. The correct corresponding authors for this Article are Yu-Chiang Chao and Cheng-An J. Lin. Correspondence and requests for materials should be addressed to Yu-Chiang Chao (ycchao@ntnu.edu.tw) or Cheng-An J. Lin (chengan_lin@cycu.edu.tw). This error has now been corrected in the HTML and PDF versions of the Article.

