# Supplementary material for: Non-Toxic Gold Nanoclusters for Solution-Processed White Light-Emitting Diodes
Source: Sci Rep. 2018 Jun 11;8:8860. doi: 10.1038/s41598-018-27201-x (PMC5995872; doi:10.1038/s41598-018-27201-x)
Supplement: Supplementary file 1 — Supplementary Information [file 41598_2018_27201_MOESM1_ESM.pdf]

Supporting Information

Non-Toxic Gold Nanoclusters for Solution-Processed White Light-Emitting Diodes

Yu-Chiang Chao[1], Kai-Ping Cheng[2], Ching-Yi Lin[2], Yu-Li Chang[2], Yi-Yun Ko[3], Tzu-Yin Hou[3], Cheng-Yi Huang[3], Walter H. Chang[3] and Cheng-An J. Lin[3]

[1]Department of Physics, National Taiwan Normal University, Taipei 11677, Taiwan.

[2]Department of Physics and Center for Nanotechnology, Chung Yuan Christian University, Chung-Li 32023, Taiwan

[3] Department of Biomedical Engineering and Center for Biomedical Technology, Chung Yuan Christian University, Chung-Li 32023, Taiwan

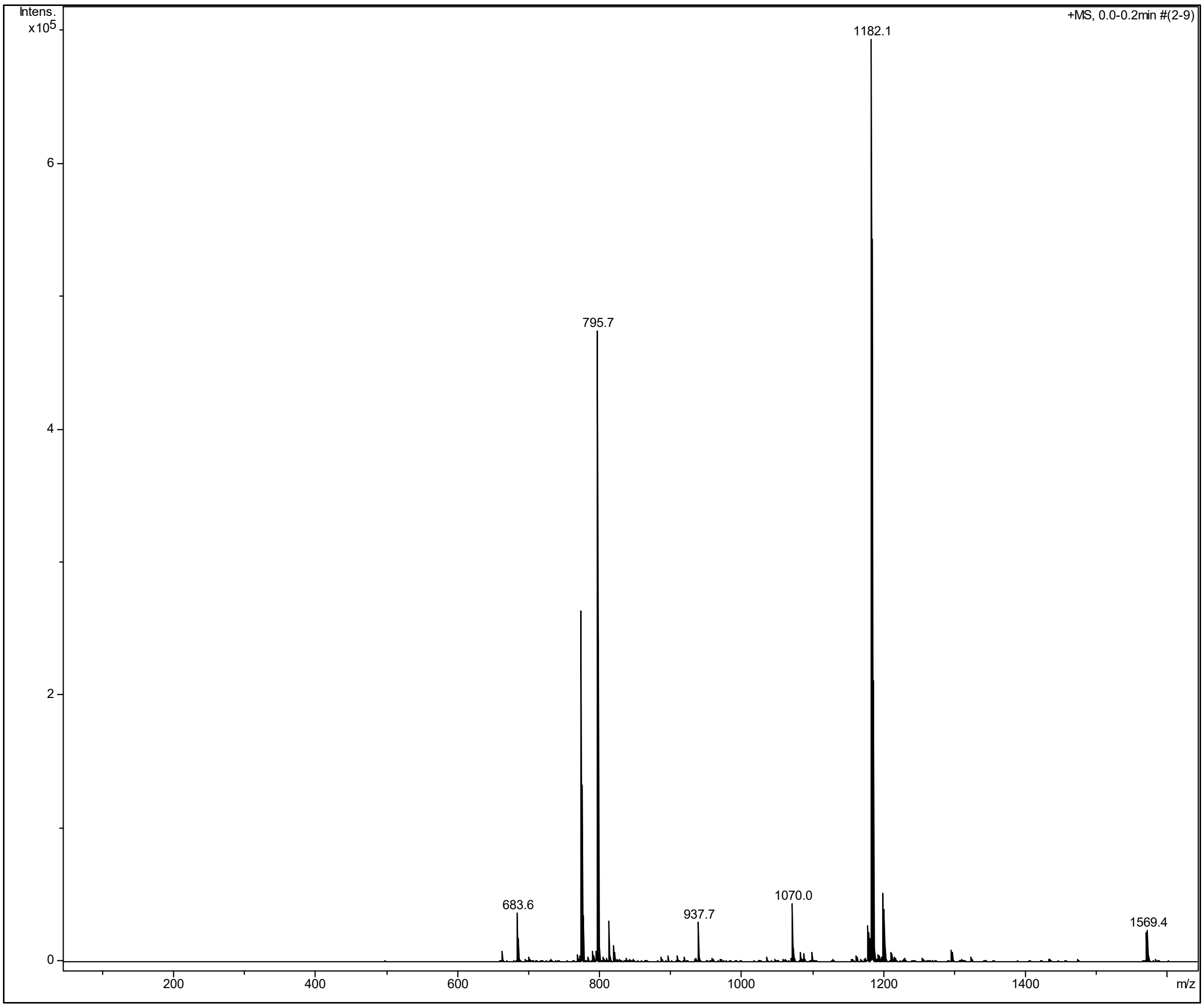

Figure S1. Mass spectra of Au NCs with TOP surface modification.

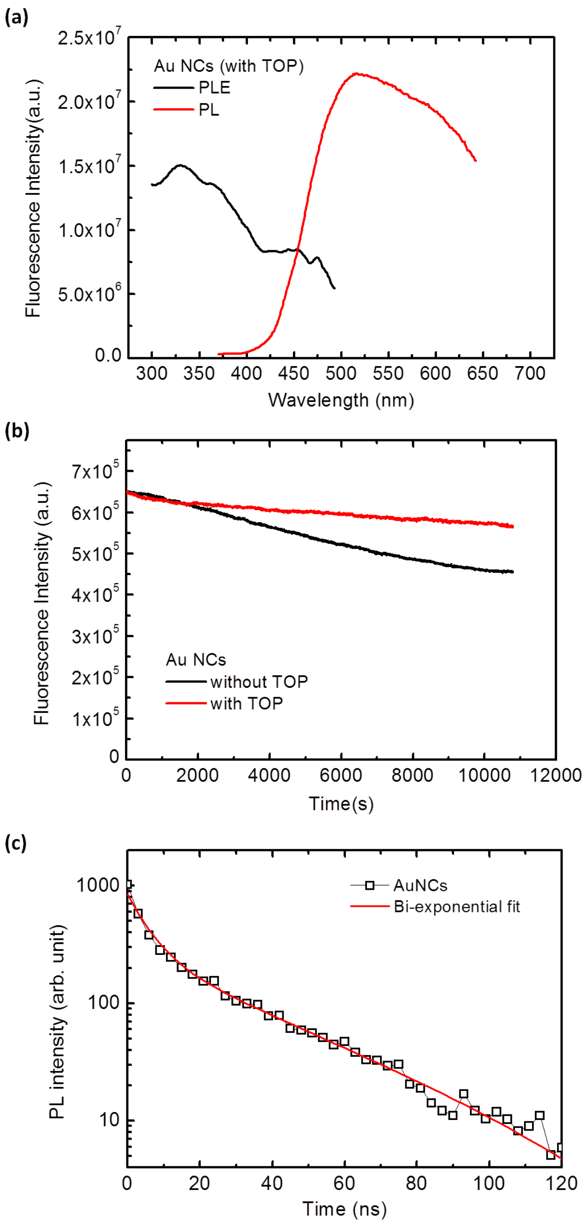

Figure S2. (a) Photoluminescence and photoluminescence excitation spectrum of Au NCs with TOP surface modification. (b) Fluorescence intensity as a function of time of Au NCs with or without TOP surface modification under UV irradiation. (c) Fluorescence lifetime of Au NCs with TOP surface modification.

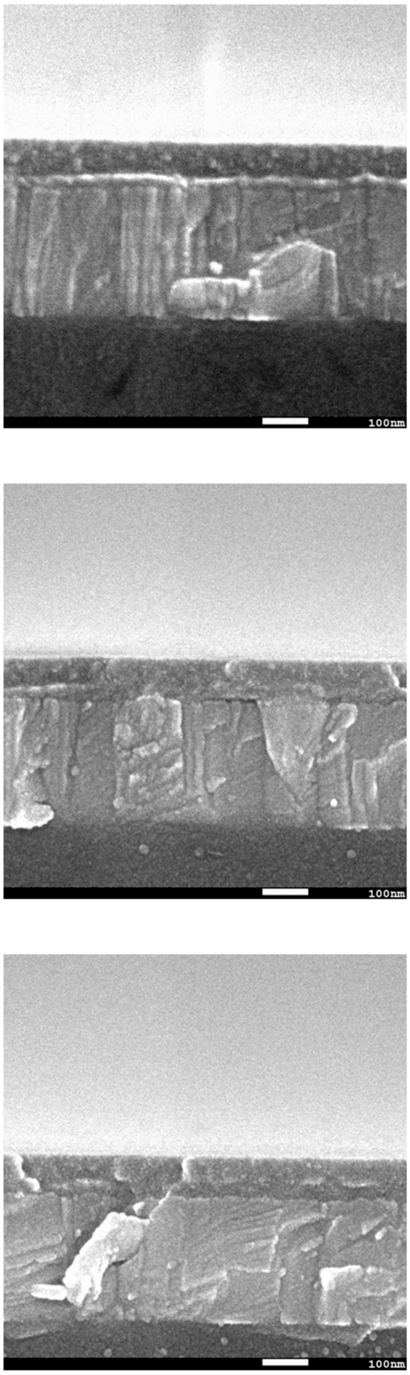

Figure S3. SEM images of the emissive layers prepared on Glass/ITO/PEDOT:PSS substrates.

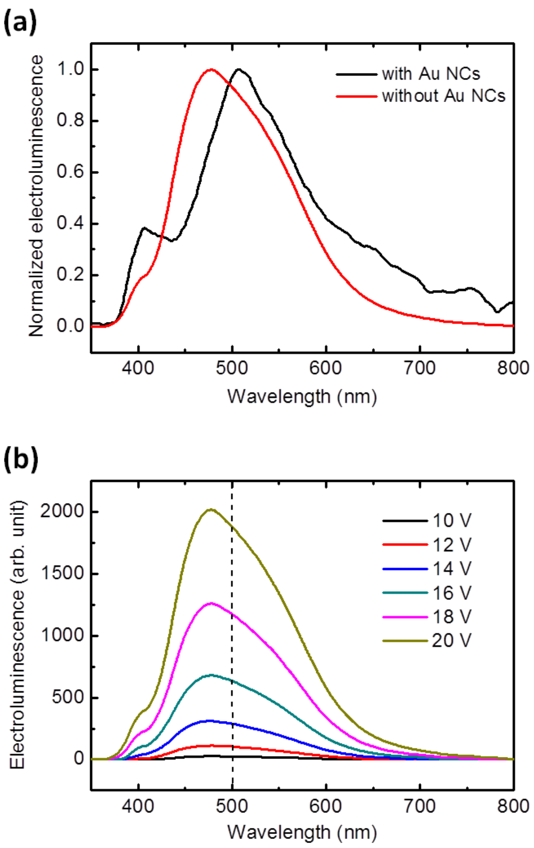

Figure S4. (a) Electroluminescence spectrum of the LEDs with and without Au NCs. (b) Electroluminescence spectra of the LED without Au NCs under various voltage biases.

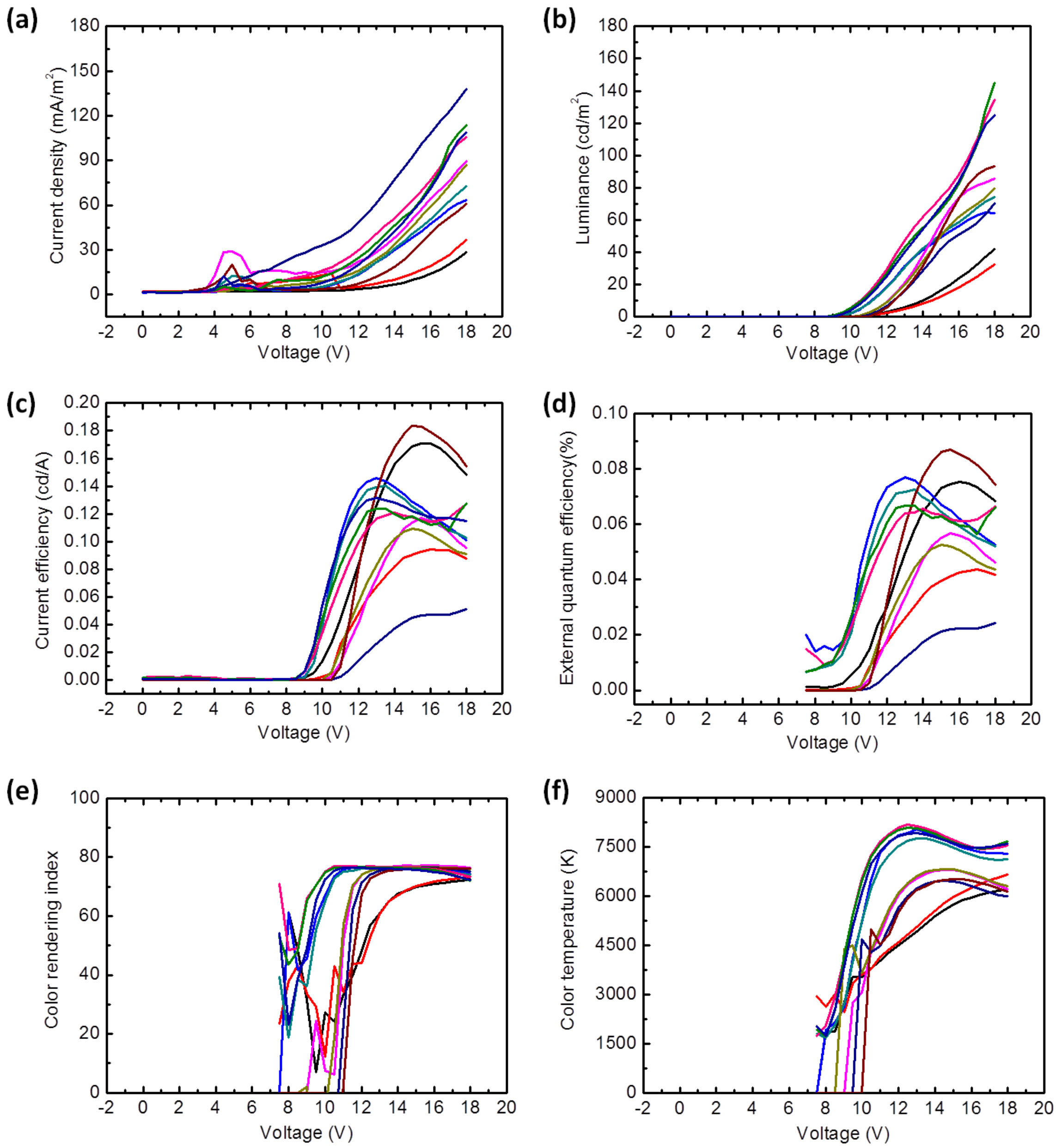

Figure S5. (a) Current density, (b) luminance, (c) current efficiency, (d) external quantum efficiency, (e) color rendering index, and (f) color temperature of eleven Au NCs LEDs.

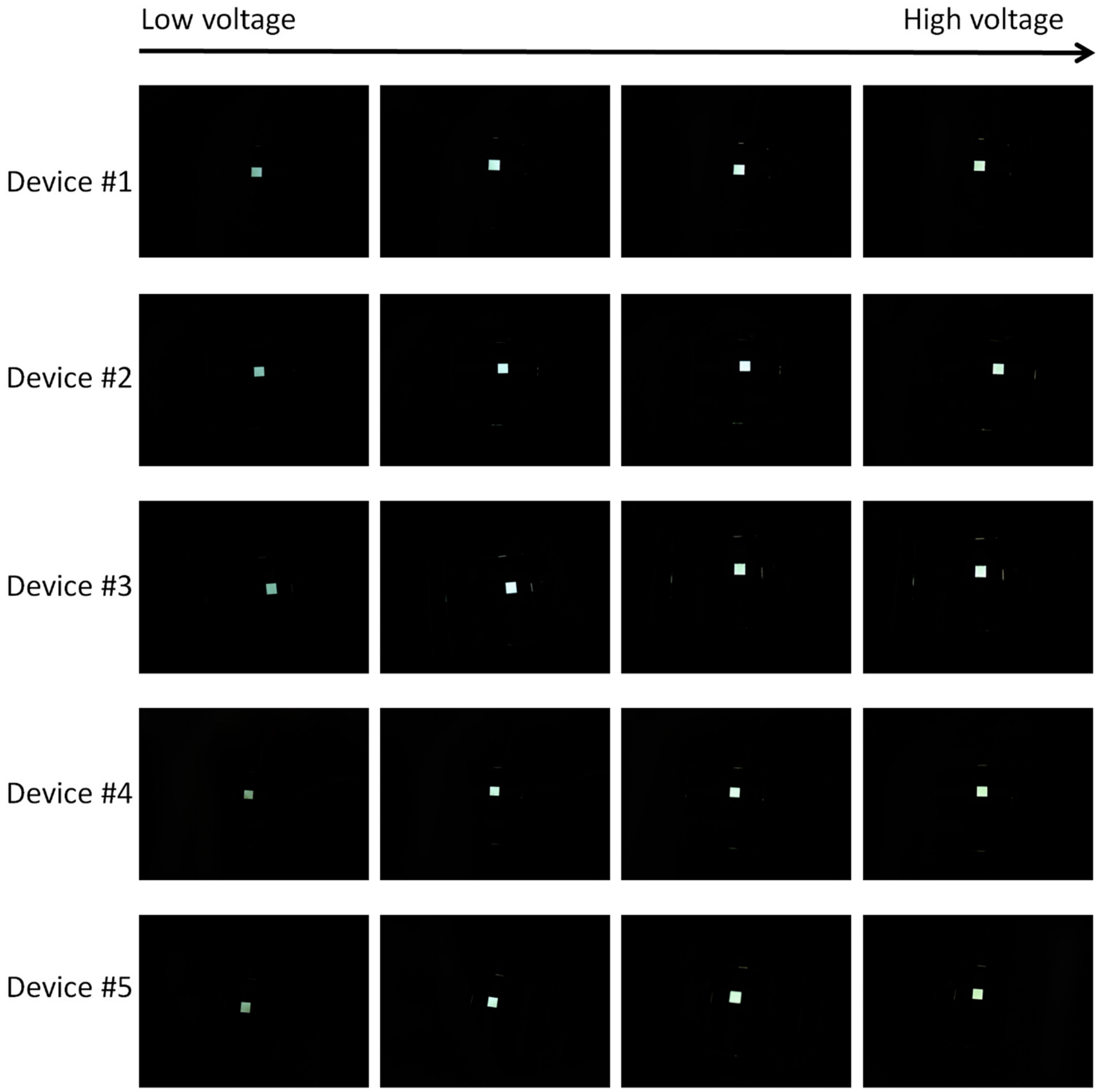

Figure S6. Photographs of five devices under various forward biases.

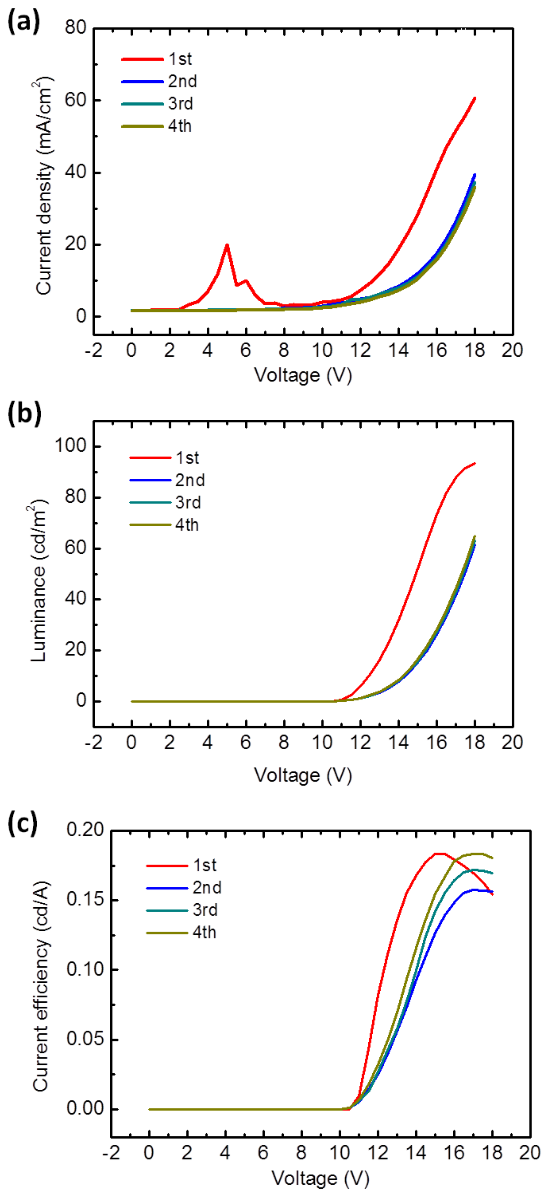

Figure S7. (a) Current density, (b) luminance, and (c) current efficiency measurements are repeated for an Au NC LED.
